# Leprechaunism

**DOI:** 10.11604/pamj.2021.40.232.31738

**Published:** 2021-12-16

**Authors:** Supraja Nagarathinam, Krishna Prasanth Baalann

**Affiliations:** 1 Department of Community Medicine, Sree Balaji Medical College and Hospital, Bharath Institute of Higher Education and Research (BIHER), Chennai, Tamil Nadu, India

**Keywords:** Elfin facies, leprechaunism, genitomegaly

## Image in medicine

Donohue syndrome is a chromosome recessive inherited disorder. It’s additionally called leprechaunism. Donohue syndrome is because of compound heterozygous or homozygous mutation within the insulin receptor (INSR) gene. It results in either complete or virtually complete absence of hypoglycemic agent receptors. Donohue syndrome is most severe type of hypoglycemic agent disorder. A 21-year-old male, born to blood- related birth-givers, could be a noted case of Donohue syndrome. He was diagnosed with this syndrome at three years of age. He was a known Type 1 diabetic with overstated hyperglycemia with hyperinsulinism and dysmorphic characteristics and cranio-facial abnormalities. Physical examination showed genitomegaly, lipoatrophy (A), abdominal distension, dermal disorders and huge depressed ears with elfin facies (B). Laboratory tests were conducted and the findings disclosed: plasma aldohexose random-339 mg/dl, blood urea nitrogen- 42 mg/dl, creatinine-3.50 mg/dl, insulin- >1000 micro-IU/ml and very low testosterone-19.82ng/dl. Clinical examinations for complete body systems were additionally performed. Chest X-ray and echo was traditional. Retinopathy screening showed macular-star in the right eye. Ultrasound abdomen showed accrued cortical echoes in each kidney with reduced corticomedullary differentiation, therefore the patient was also on weekly chemical dialysis. Neurological examinations were intact. Deoxy ribonucleic acid analysis using period-time polymerase chain reaction (PCR) are frequently used for distinguishing this syndrome. Other diagnosis are Type-A syndrome and Rabson Mendenhall syndrome. Treatment needs efforts from endocrinologists, dermatologists and numerous different medical professionals. Treatment with insulin-like protein one can also be contemplated.

**Figure 1:**
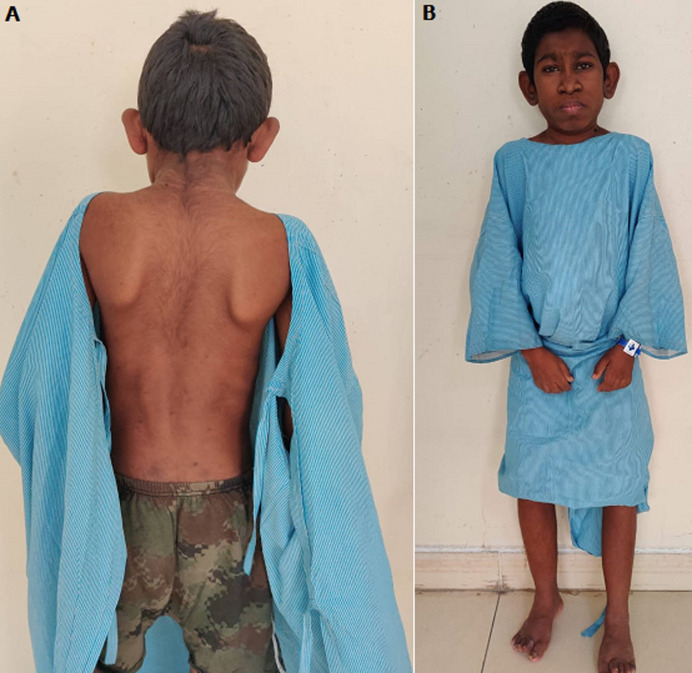
(A) lipoatrophy; (B) huge depressed ears with elfin facies

